# Symptoms of Post-Traumatic Stress Disorder and the Sense of Gains and Losses during the COVID-19 Pandemic: An International Study

**DOI:** 10.3390/ijerph19063504

**Published:** 2022-03-16

**Authors:** Ewa Małgorzata Szepietowska, Ewa Zawadzka, Sara Filipiak

**Affiliations:** Department of Clinical Psychology and Neuropsychology, Institute of Psychology, Maria Curie-Sklodowska University, 20-612 Lublin, Poland; ewa.szepietowska@mail.umcs.pl (E.M.S.); ewa.zawadzka@mail.umcs.pl (E.Z.)

**Keywords:** COVID-19 pandemic, post-traumatic stress disorder, posttraumatic stress disorder, changes of life, sense of gains and losses

## Abstract

This study was primarily designed to investigate the perception of changes in selected areas of life experienced by adults of various nationalities in connection to the long-lasting COVID-19 pandemic. The second objective was to identify the factors increasing the risk of perception of negative changes in life during the pandemic. The tools applied in the study include a self-report questionnaire designed to measure sociodemographic data and health status of the subjects, COVID-19 Sense of Life Changes Questionnaire, as well as the Impact of Event Scale: Revised. The study involved over 600 adult subjects. With increased intensity of intrusions, the likelihood of negative perception of the changes emerging as a result of the pandemic was reduced by approximately 7%, whereas a higher intensity of hyperarousal increased that risk. Individuals reporting a sense of negative changes presented a greater degree of hyperarousal compared to those reporting positive changes. In the group of subjects perceiving the changes in a positive way, increasing the intensity of intrusion and/or hyperarousal corresponded to a growing conviction about a negative nature of life changes concerning the relationship with their partner and affecting their work as well as regarding a positive meaning of the changes in relations with their parents and in daily life. Generally, there was a prevailing sense of negative changes; however, there was also a group of subjects that perceived these as positive. Intrusions and hyperarousal in certain individuals may play a role in motivating them to take action in protecting against effects of the pandemic and, in others, may lead to frustration and anxiety.

## 1. Introduction

From 2019, the world has been coping with COVID-19 pandemic induced by SARS-Cov-2, a virus from the group of coronaviruses. Towards the end of July 2021, the cumulative numbers of cases and deaths reported globally were almost 194 million and over 4 million, respectively [1]. In December 2021, the cumulative number of cases reported globally exceeded 272 million, and the cumulative number of deaths exceeded 5.33 million [2]. At the time the study was conducted, the increase in the incidence (fourth and fifth waves of the pandemic) was associated with the predominant Delta variant [3,4] with less common cases induced by Omicron variant [5].

Although the pandemic is a natural disaster, like floods and fires, the world has not encountered such widespread suffering, anxiety and uncertainty since the mid-twentieth century [6,7]. Beyond the direct impact of COVID-19 itself on physical health, the situation has also negatively impacted people’s life in all of its aspects [8]. The negative consequences of the successive waves of the pandemic have affected many areas in our lives and those of many communities.

Due to this we speak of worldwide sense of total trauma and total loss [9]. The sense of loss during the relevant period took on many faces, ranging from severe trauma during the first wave of the pandemic to multiple simultaneous losses or compounded loss [10] or a*mbiguous* loss [11]. Ambiguous loss is any type of loss that is unclear and therefore has no closure. It can be described as a situation of experiencing the feelings of grief without knowing exactly what or why we are grieving. 

The faces of trauma and sense of loss throughout the duration of COVID-19 pandemic have been impacted by an interaction of many factors, such as the sudden onset of the pandemic; information about the scale of its spread and the numbers of patients and deaths; lack of clarity regarding the source of infection, information related to the symptoms, and methods of preventing infection; as well as lack of information about treatment options [12,13]. 

Trauma was induced by the lockdowns introduced in successive countries, periods of withdrawal from or reinstating restrictions, the uncertainty related to the introduced vaccines and post-vaccination symptoms, and currently the uncertainty about the future in connection to the spreading infections with Delta and Omicron variants [14]. Research findings from 2019–2020 reported an increased sense of threat to life, and to motor and cognitive abilities, both in patients and in entire communities [15,16], increased incidence of complicated grief in connection to the death of the loved ones and the inability to be present in the last moments of their life [17] as well as loss of rituals (weddings and religious ceremonies), loss of work and financial status, loss of social support and sense of security, and even loss of one’s sense of identity [7,18,19].

The set of symptoms linked to the pandemic (*Post-COVID Stress Disorder*) matches the criteria defined for PTSD and comprises hyperarousal (increased reactivity to stimuli), avoidance (symptoms manifested in evading stimuli associated with the event), intrusions/ruminations (focusing on the negative aspects of the situation, incessant and passive contemplation of negative events (which aggravates the sense of helplessness and depression) [20]), as well as the dysphoric and anxious arousal associated with memories of the COVID-19 pandemic or related events [21,22]. 

A high frequency of post-COVID PTSD was observed in patients with a moderate-to-severe course of COVID-19 [19,23,24,25] and in individuals with other diseases [26]. Symptoms of PTSD were also identified in individuals who had not suffered from COVID-19 [18,27], in health care workers [28,29,30], young adults [31], and senior citizens [32].

Experiencing PTSD in connection to COVID-19 may also play a role in the development of psychopathological symptoms and delayed symptoms (continuous traumatic stress) [33]. A study by Ye et al. [34], involving Chinese students, showed an association between intensity of rumination and depression, and demonstrated a role of rumination in the experience of stress due to the pandemic [35]. Intrusive ruminations contributed to a feeling of psychological distress [36,37]. A study from Slovakia showed that rumination in connection to anticipated loss of income, insufficient opportunities to talk with others, and a subjective sense of isolation, low tolerance of uncertainty as well as catastrophizing, analysed as psychological variables, decreased the frequency of positive experiences and increased the frequency of negative emotions and severity of depression [38].

Unlike other stressful situations, PTSD following COVID-19 is not only related to the past but also to the future. Anticipatory loss refers to anticipated death of a loved one, e.g., due to COVID-19 [39]; however, the term may also be related to any expected changes in all the areas of life and economy and in one’s own life [40]. 

The changes that took place during the pandemic and those that are still predicted to happen are also the cause of ambivalence in emotions, attitudes, and behaviours. The situation during the pandemic (lockdown and on-line education) produced both negative and positive emotions. This tendency was shown by a study involving Italian adolescents [41] who, in their narratives, pointed out negative and positive experiences during the pandemic, e.g., they had an opportunity to “re-discover their families”. 

A longitudinal study by Chen et al. [42] showed that, during the COVID-19 pandemic in 2020, a change was observed amongst Chinese people in their understanding of the source of meaning in life. The respondents attached less importance to personal accomplishment and social status, as well as religion and enjoyment, and they admitted a growing need for a simple and balanced lifestyle and for contributing to their community. Findings by Cox et al. [43], acquired in a study involving 451 students, showed a sense of growth in relationship investment, gratefulness, and patience. 

On the other hand, Wong [44] found that Chinese adults in Hong Kong reported both gains and losses with regard to family relationships and mental health, resulting from the pandemic; these were largely affected by socioeconomic status. Increases in the knowledge related to the pandemic and awareness of health promoting behaviours were also emphasized [45].

Experience of trauma, post-traumatic response, ways of coping with trauma, as well as opinions about the impact of trauma on one’s own life are shaped by the cultural background and the related cultural experiences as well as the political and economic situation of a country [46]. A study by Ruiz et al. [47], which assessed 1131 individuals from the United Kingdom, South Korea, Finland, Philippines, Latin America, Spain, North America, and Italy, showed that residents of those countries reported various degrees of well-being during the COVID-19 pandemic. 

Data reported by Pauw et al. [48] from 23,865 respondents from 51 countries showed that ruminations and suppression were predictors of poorer mental condition; cultural affiliation (investigated in the categories: individualism—collectivism) did not moderate relations between the subjects’ wellbeing and rumination or suppression. On the other hand, a study of Poles and Ukrainians conducted by Długosz et al. [49] showed that, irrespective of the individual’s nationality, better adjustment to the situation of lockdown was determined by financial status and level of education. 

Other studies [50,51] confirmed that a number of variables linked to nationality and country of residence affect the severity of PTSD and behaviours during the COVID-19 pandemic (socioeconomic status; intensity of fatalistic beliefs; attitude towards restrictions, etc.). No relationship between culture and psychological distress, depression or rumination was identified [52].

Longitudinal studies covering the years 2019–2021 and a few waves of the pandemic point to two phenomena that are important for our study. One of these relates to adaptation to the current situation observed in various populations despite the existing pandemic [53,54]. Effects of adjustment to “the new normal” include decreases in negative emotions as well as the characteristic features of post-traumatic growth, such as tendencies for increased reflectiveness, greater interest in spirituality and nature, and deeper relations with others [55]. 

Another issue is the relation between the adaptation process and individual or external characteristics [56,57]. The development and persistence of post-COVID PTSD are promoted by individual socioeconomic and psychological factors, such as unemployment, isolation [18], younger age, being a woman, lower level of education, being single, staying with more children, living in a country or area more severely affected by COVID-19 [56,58], obsessive thinking about COVID-19, and anxiety caused by COVID-19 [59].

In view of the dynamic evolution of the pandemic situation (the existing fourth wave and/or a risk of the fifth wave of infections), in the present study, we aimed to investigate the sense of changes in various areas of life, experienced by individuals of various nationalities in connection to the long-lasting COVID-19 pandemic. The study was also designed to identify the characteristics of individuals reporting negative and positive changes in various spheres of life resulting from the pandemic and to determine individual and psychological factors that may predict a greater sense of negative changes in connection to the earlier waves of COVID-19.

## 2. Materials and Methods

An online survey, intended for adults, was performed from 1 September to 12 December 2021, with the use of Google Forms (Google Poland, Lublin, Poland). The snowball method was applied in collecting data. General information about the purpose of the study was sent out via private e-mail and Facebook (Facebook Poland, Warsaw, Poland), with a link to demographic survey questions and to the questionnaires. The research tool was prepared in accordance with the recommended standards for conducting and reporting web-based surveys (CHERRIES). 

Potential participants were informed that submitting the completed online questionnaire was equivalent to giving consent to participate in the study. They were also informed that their responses would be used for research purposes only. All the tools were prepared in three language versions, i.e., Polish, Ukrainian, and English. Over 600 individuals of various nationalities participated in the survey. Due to the fact that some questionnaires were not fully completed, the analyses eventually considered data related to 492 participants.

We developed a self-report questionnaire to collect demographic (nationality), personal (gender, age, financial status, education, and employment situation), and medical data (health condition, COVID-19 vaccination, personal history of COVID-19, and COVID-19 in relatives) as well as COVID-19 Sense of Life Changes Questionnaire a COVID-19 (COVID-19 SLCHQ).

In COVID-19 SLCHQ, the respondents were asked whether and in what way (negative, positive, or neither) the COVID-19 pandemic had changed their life and its various aspects. They were asked to consider the years 2020 and 2021. The items (a total of 17) referred to issues, such as relations with children and partners, finances, work, religion/spirituality, social activity, etc., and the final statement (17) was related to opinions about the future. 

The respondents were asked to assess the changes in the various aspects of life, by selecting one of the responses on a 7-point scale, where 1 = dramatically negative, 2 = very negative, 3 = rather negative, 4 = neither negative nor positive, 5 = rather positive, 6 = very positive, and 7 = extremely positive. Hence, a higher score reflected an opinion that the changes that occurred during the COVID-19 pandemic were positive, and a lower score corresponded to an opinion that the changes were negative. Option 0 = not applicable was used to account for individual differences (e.g., no children, no partner, etc.).

Severity of PTSD was measured using the Impact of Event Scale: Revised (IES-R) [60,61] in the respective language versions. Internal consistency of the Polish version of IES-R (Cronbach’s alpha) was 0.92, and the respective values of the coefficient for hyperarousal, intrusion, and avoidance were as follows: 0.89, 0.85, and 0.78 [61]. The correlations between the general score in the scale and most items were reflected by values exceeding 0.60. 

Weiss and Marmar [60] reported very high internal consistency of the three subscales with intrusion alphas in the range of 0.87–0.92, avoidance alphas in the range of 0.84–0.86, and hyperarousal alphas between 0.79 and 0.90. In the Ukrainian version, Cronbach’s alpha for the total measure in the sample of 188 Ukrainian service members was 0.90. In this study, the reliability co-efficient for total sample was 0.89 (total IES-R), 0.87 (intrusion subscale), 0.86 (hyperarousal subscale) and 0.79 (avoidance subscale). The respondents were asked to consider the years 2020 and 2021.

The IES-R is a self-administered, 22-item questionnaire covering three clusters of symptoms identified in the Diagnostic and Statistical Manual of Mental Disorders, third edition (DSM-III), as indicators of post-traumatic stress disorder (PTSD): Intrusion (eight items on the scale), Avoidance (eight items on the scale), and Hyperarousal (six items on the scale). Intrusion is characterized by nightmares, unbidden visual images of the traumatic event or its aftermath while awake, intrusive thoughts about aspects of the traumatic event, sequelae, or self-conceptions. Avoidance is typified by deliberate efforts to not think about the event, to not talk about the event, and to avoid any reminders of the event. 

Equally characteristic are more active attempts to avoid memories and recollections of the event or its aftermath by increasing use of alcohol or drugs, overworking, or by using other strategies designed to divert attention or to exhaust someone so that he or she is temporarily untouched by the intrusive phenomenology. The hyperarousal scale covers factors, such as anger, irritability, hypervigilance, difficulty concentrating, and heightened startle. Those assessed with the IES-R were asked to rate their feeling of distress with regard to 22 symptoms, according to a five-point scale: 0 = not at all; 1 = a little bit; 2 = moderately; 3 = quite a bit; and 4 = extremely. 

The total score for each subscale was calculated using the mean of the scored responses. The raw scoring is in the range from 0 to 88 points. A score of 24 or more points mean that PTSD is a clinical concern. Those with such high scores who do not have full PTSD may have partial PTSD or at least some of the symptoms. A score of 33 points or more represents the best cut-off for a probable diagnosis of PTSD, a score of 37 or more is high enough to suppress the person’s immune system functioning (even 10 years after an impact event).

Survey data were exported to Excel (Microsoft, Lublin, Poland) from Google Format initially and were then transferred to SPSS version 26 ( IBM SPSS Statistics delivered by Predictive Solutions, Lublin, Poland). Participants’ characteristics contain data related to frequency, and percentages and—in the case of quantitative data—the means (M) and standard deviations (SD). A Pearson’s chi-squared test of independence was performed to compare categorical variables in the two groups. Due to the approximately normal distribution of the variables, comparative assessment of independent quantitative data was conducted using Student’s t-test (for two groups) or One-way ANOVA and post-hoc comparison with Bonferroni correction (for three groups). 

Comparative analysis of the dependent data (IES-R sub-scales) was conducted using a dependent (paired) Student’s t-test. Binomial logistic regression model (enter method with likelihood ratio) was created to evaluate factors possibly related to the sense of negative life changes caused by the pandemic (dependent variable encoded as follows: negative changes: 1; positive: 0); independent variables: demographic and psychological characteristics. Pearson’s r correlation coefficients (one-sided) were used in the analysis of the relationship between IES-R and COVID-19 SLCHQ. A significance level of *p* ≤ 0.05 was adopted in all analyses.

The study was conducted according to the guidelines of the Declaration of Helsinki. The design of the study was approved by the local Research Ethics Commission (protocol code 8/2021).

## 3. Results

### 3.1. Characteristics of Participants

The respondents ranged in age from 17 to 70 years (mean = 34.14 ± 13.17 and median = 32.0). Detailed socio-demographic characteristics of the sample are provided in Table 1. The majority of the respondents were from Europe. The group predominantly included young adults, females, working people/university students, and individuals sharing their place of residence with other people (partners and parents). The majority of the respondents reported they had not suffered from COVID-19 and that they had been fully/partly vaccinated against COVID-19. The individuals who had recovered from COVID-19 as a rule had not needed hospitalization. Similar numbers of respondents reported cases of COVID-19 disease, or no such incidents in the family. The majority of the respondents reported good health and financial status similar to that of other people in their communities.

### 3.2. Severity of PTSD and Sense of Life Change Due to the Pandemic

The results of all the respondents in IES-R and COVID-19 SLCHQ were calculated at the first stage of the analyses. The related data are shown in Table 2 and Table 3. The cumulative mean score in IES-R matches the cut-off point (24 point) and is indicative of PTSD symptoms. The symptoms of avoidance and hyperarousal were more severe, whereas the signs of intrusion were less severe. Statistically significant differences were observed (Intrusion—Hyperarousal *p* = 0.001; Intrusion—Avoidance *p* = 0.001; and Hyperarousal—Avoidance *p* = 0.001). 

In some of the 17 areas included in COVID-19 SLCHQ, the respondents reported no change or positive change (relations with parents, children, or grandchildren, opportunity to cultivate a hobby, and religion), the highest score reflecting the respondents’ opinion about greater attention to health. Notably, however, some participants did not give responses to six items (see Table 3). The respondents also reported negative changes in their daily life, activity for others, mental and physical health, as well as finances. 

As some items did not apply to some of the respondents (e.g., those having no children or following no religion), the overall COVID-19 SLCHQ index was calculated considering 11 out of 17 items (100% responses) (see Table 3). It was possible to acquire a score in the range of 11–77 points; lower scores corresponded to stronger opinions about negative changes in various spheres of life due to the pandemic and higher scores reflected stronger belief about positive changes. The cut-off point was the score of 44—the respondents’ scores on average were below this limit (mean = 40.75). 

Gender, personal experience of COVID-19, as well as the health status of relatives did not differentiate the scores in IES-R or in COVID-19 SLCHQ. Compared to the respondents who had been fully or partly vaccinated, those who declared no intention to become vaccinated were more likely to show significantly stronger avoidant tendencies. Respondents with lower education were found to have a higher degree of hyperarousal, whereas those with higher education were more likely to acknowledge beneficial changes resulting from the pandemic (Table A1).

### 3.3. Characteristics of Participants with a Sense of Positive and Negative Changes in Life as a Result of the COVID-19 Pandemic

**Figure 1:**
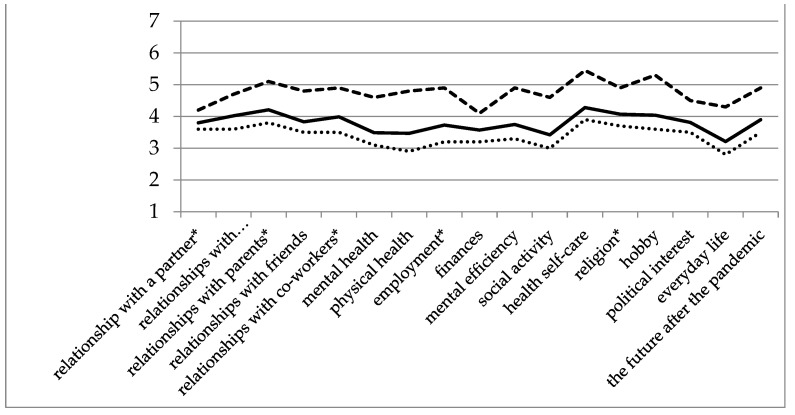
Sense of changes in various areas of life in the negative (NCH) and positive (PCH) sense of changes groups (solid line—the whole sample; dotted line—NCH group, and dashed line—PCH group); * scales not included in COVID-19 SLCHQ.

In the next part of the analyses, the k-means method was applied to determine two groups of respondents based on the cumulative score in COVID-19 SLCHQ; one group comprised those with a sense of negative changes resulting from COVID-19 pandemic (sense of negative changes in life –NCH group, *n* = 362), and the other consisted of those presenting opinion about positive changes in various areas of life (sense of positive changes in life—PCH group, *n* = 130) (detailed data—Table 4). The cumulative score in COVID-19 SLCHQ significantly differentiated the groups (NCH: M = 36.3 ± 6.66, PCH: M = 55.08 ± 7.0, t = 24.25, *p* = 0.001). The NCH group was nearly three-times larger than the PCH group. The groups did not differ in terms of the demographic or medical data; however, they were found with a different degree of hyperarousal—the NCH group had higher score; however, the size of the effect (Cohen’s d) was low. The sense of changes in life in each group are shown in Figure 1.

### 3.4. Relationships between IER-S Scales and the Sense of Changes in Life Caused by the Pandemic in the NCH and PCH Groups

As the groups differed only in the intensity of hyperarousal, analyses were performed to assess Pearson’s r correlation coefficients for IES-R and the subscales as well as the COVID-19 SLCHQ scores separately for each group (NCH, PCH). Table 5 presents data for statistically significant correlations only. A number of significant relationships between these variables were found in the NCH group; in all cases, the correlations were negative, and they were connected with many areas of life (mental and physical health, work, finances, intellectual capacities, activity for others, opportunity to cultivate a hobby, attention to politics, and daily life). 

A higher degree of intrusion, avoidance, and hyperarousal corresponded to a growing sense of negative changes in these areas of life. Fewer significant correlations were found in the PCH group. Negative associations were found between PTSD and the relation with a partner as well as one’s work; a higher degree of intrusion and/or hyperarousal coincided with stronger opinion about negative character of changes in these areas of life due to the pandemic. 

Unlike in the NCH group, findings in the PCH group showed significant positive associations, i.e., a higher degree of intrusion corresponded to more positive opinions about relations with parents during the pandemic, and a higher degree of intrusion and hyperarousal as well as higher overall IES-R score coincided with more positive opinion about changes in one’s daily life. These positive correlations differentiated the PCH group from the NCH group.

### 3.5. Determinants of the Sense of Negative Life Changes Caused by the Pandemic

Logistic regression was calculated in order to identify factors increasing the risk of negative perception of changes in life resulting from the pandemic (Table 6). Classification within the groups with different perception of the changes due to the pandemic was adopted as the dependent variable (the coding applied: NCH = 1 and PCH = 0), and the following predictors were defined: respondents’ age, gender, education, occupational involvement, personal experience of COVID-19, COVID-19 in one’s family, vaccination status, IES-R avoidance, intrusion, and hyperarousal. 

One model was obtained that effectively matched the data (χ^2^ = 18.773, *p* = 0.03; −2 log-likelihood = 547.57, Nagelkerke’s R^2^, *p* = 0.06; Cox, Snell R^2^, *p* = 0.07; Hosmer and Lemeshov test χ^2^ = 9.268, *p* = 0.320, % of correct classifications 73.2). The following factors were excluded from the model: age, level of education, avoidance, gender, type of occupational involvement, vaccination status, health status of relatives in connection to COVID-19, and personal experience of COVID-19. With an increase in the intensity of intrusion, there was an approximately 7% decrease in the likelihood of negative perception of the changes due to the pandemic, whereas a greater degree of hyperarousal increased that risk by approximately 16%. 

The other variables were not found to significantly predict a greater risk for negative perception of the changes resulting from the pandemic. Notably, however, age and education level potentially play a role in increasing the likelihood of negative perception of changes due to the pandemic. With age, the likelihood of negative perception of changes due to the pandemic increased slightly. Analysis of Pearson’s correlation coefficients showed that beliefs about negative change in relations with parents (r = −0.127, *p* = 0.005) and attention to health (r = −0.07, *p* = 0.05) increased with age. Likewise, lower education potentially is a risk factor for negative perception of changes due to the COVID-19 pandemic. 

Higher education level was associated with reduced beliefs about negative changes in life during the pandemic. Compared to individuals with higher education (hl—higher level) those with lower education (ll—lower level) were significantly more likely to have negative perceptions of their relations with partner (3.55 ± 1.64_ll_ vs. 3.97± 1.45_hl_ t = −2.54 *p* = 0.012), their mental health (3.34 ± 1.20_ll_ vs. 3.60 ± 1.27_hl_ t = −2.12 *p* = 0.034), work (3.27 ± 1.77_ll_ vs. 3.95 ± 1.46_hl_ t = −3.83 *p* = 0.001), finances (3.41 ± 1.46_ll_ vs. 3.68 ± 1.29_hl_ t = −2.154 *p* = 0.03), intellectual capacities (3.57 ± 1.47_ll_ vs. 3.88 ± 1.28_hl_ t = −2.54 *p* = 0.012), activity for others (3.11 ± 1.43_ll_ vs. 3.64 ± 1.35_hl_ t = −4.21 *p* = 0.001), attention to health (4.04 ± 1.50_ll_ vs. 4.45 ± 1.42_hl_ t = −3.002 *p* = 0.003), and future after the pandemic (3.69 ± 1.60_ll_ vs. 4.06 ± 1.41_hl_ t = −2.67 *p* = 0.008).

## 4. Discussion

The primary purpose of the study was to determine in what way changes in various areas of life resulting from the current pandemic were perceived by people. The study involved individuals representing various nationalities. Generally, respondents’ opinions reflected negative perception of these changes; yet, this did not apply to all areas of life. Changes in attention to health and the opportunity to cultivate a hobby were indicated as those perceived in a positive way, while negative changes, according to the respondents, affected daily life, activity for others, as well as mental and physical health and finances. Similarly, changes affecting one’s religion or relations with children/grandchildren were also perceived as positive, although this only applied to respondents who completed the relevant parts of the questionnaire. 

Most generally, the findings to a degree reflected a sense of change in life; however, on average, these changes were not perceived by the respondents as dramatically adverse or extremely beneficial. This clearly reflects the changes that have taken place in our thinking about the effects of trauma that has continued for nearly two years [55]. The early research (2019–2020) emphasized a sense of loss caused by death of loved ones [9], restrictions leading to a sense of isolation [62], or from financial damages [44,63], as well as inability to meet professional obligations, cf. research referred to in [64]. 

Other studies reported increased frequency of unhealthy behaviours and domestic violence [65] as well as increased distress [51,53,66]. On the other hand, a growing importance of religious practices was reported by Bentzen in a study conducted in 95 countries [67]. Other studies emphasized the importance of interpersonal relationships and their protective role [55]. The first waves of the pandemic caused a sense of multiple simultaneous losses or compounded loss [10,18], or a*mbiguous* loss [11]. Other researchers demonstrated the presence of a day-to-day carry-over effect of negative mood and anxiety related to pandemic, feeling confined by COVID-19, and increased negative mood in daily life [68]. 

Retrospective and longitudinal studies conducted in 2021 demonstrated a gradual mild decrease in anxiety. An important role in impacting mental condition was played by the perception of gains (e.g., better knowledge of preventive measures reduced the severity of anxiety) and losses as a result of the pandemic (e.g., negative emotions were related to the loss of job or more frequent family conflicts) [43,69]. Decreased anxiety may be attributed to the phenomenon of habituation and becoming used to the situation, which was no longer new and unpredictable. 

The mechanism occurs jointly with simultaneously growing fatigue and a sense of excessive stimulation (overdose). The phenomena associated with habituation during the pandemic were observed in Germany [70] and Japan [71] at the time of the successive waves. The observed effects included a lower sense of loss of work-related opportunities in scientists [72] and greater interest in physical activity possible despite the restrictions and attempts to perform more exercise [73]. 

The dynamic perception of trauma due to the pandemic is discussed in terms of resilience [74]. Having a sense of meaning in life is treated as a protective factor in the currently experienced crisis, cf. [8]. Galatzer-Levy et al. [75] point out that resilience is the most common condition leading to recovery of balance following trauma. The trajectory of changes includes not only resilience but also chronic state of PTSD and asymptomatic or delayed onset [76]. This diversity of responses suggests that the sense of losses or gains due to the pandemic is heterogenous.

The second purpose of the study was to determine the role of demographic variables and severity of PTSD in the changing perception of losses or gains. Participants of the study, as a group, acquired a borderline score in IES-R (cut-off 24/88 points). The scores reflect higher avoidance and hyperarousal and lower intrusion. Similar findings were reported by studies that involved individuals experiencing the Ebola epidemic [77] and the current pandemic [78]. Interestingly, individuals with a sense of negative and positive changes in various areas of their life differed only in the intensity of hyperarousal. However, the associations between the severity of PTSD and the perception of changes resulting from the pandemic were different in the groups. 

A sense of loss in many areas of life was found to be significantly related to higher avoidance, hyperarousal, and intrusion in individuals perceiving the changes as negative. On the other hand, in the group of individuals reporting positive changes, there were few significant correlations between the variables; a higher degree of intrusion and hyperarousal corresponded to positive perception of the changes in the daily life and in relations with parents during the pandemic. 

Notably, individuals perceiving changes in a positive way were likely to more effectively adapt to the pandemic situation. Despite, or owing to adversities, they were able to notice what is valuable for the experienced quality of life, for their mental health and well-being. Perhaps they use some creative or existential strategies, enabling transformation of anxiety, fear, or adversity into positive thoughts and personal growth; this may facilitate their functioning during the successive waves of the pandemic and coping with the related restrictions. It may also promote acquisition of new resources that can be applied to deal with crisis events. This may be gained by adopting the attitude of challenge to protect against COVID-19 and/or accepting the harshness of the situation or appreciating the meaning in life [79]. 

The efficacy of coping mechanisms depends not only on the nature of the traumatic situation but also on the individual and cultural factors, see: [8] for details. By considering PTSD as a factor potentially contributing to negative perception of changes in various areas of life, it was possible to identify the significant, yet different role of intrusion and hyperarousal. Higher hyperarousal was a risk factor for greater sense of loss due to the pandemic whereas intrusions decreased that risk slightly. This finding is different than the results acquired in a study by Sanchez-Gomez [78], where greater intrusion resulted in higher hyperarousal, which adversely affected the perceived mental health. 

We presume that this unexpected effect of intrusions may be associated with their characteristics: they may relate to memories (intrusive memories) and thoughts (intrusive thoughts) [80]. Intrusive thoughts are linked to anxiety, which, during the pandemic, affected whole communities. However, during the entire period of the pandemic, we learned to continuously think about COVID-19, i.e., follow media reports, apply the required safety measures, maintain relations with and take care of friends and family, work and study online; perhaps anxiety determines intrusive thoughts and behaviours that lead to a sense of security and sense of control over events, which, in turn, reduces the subjective sense of losses suffered. 

Other researchers also reported evidence suggesting a perception of positive changes, e.g., increased attention to and more frequent contacts with relatives, beneficially affecting perception of one’s relationship with them SARS—[81], as well as the feeling of slowing down the pace of daily life [82]. Intrusions may have contributed to the routinisation of daily life, which produces a sense of normality, control, and predictability; this may foster a sense of positive changes despite the pandemic [83]. Notably, however, hyperarousal adversely affects self-perceived quality of life [78,84]. 

On the one hand, the current pandemic demands vigilance in everyday situations, and on the other hand, this constant monitoring increases the sense of uncertainty and loss. It has also been shown that the impaired self-perceived quality of daily life during the pandemic exacerbated the signs of PTSD [63]. A sense of security in some individuals, generated by excessive vigilance and cautiousness, may be superficial when behaviours are fundamentally affected by anxiety [85]. In summary, the present findings suggest that intrusions and hyperarousal may play a different role in affecting the sense of loss or gain during the pandemic.

The sense of gains and losses resulting from the pandemic were also significantly affected by some demographic factors. With age, there may be a growing sense of negative changes due to the pandemic. Research reports have shown a positive relationship between age and COVID-19 anxiety [74]; however, it was also found that age corresponded to perception of gains resulting from the pandemic in various areas of life [44,56]. The present findings support the above conclusions since the sense of loss was related only to two areas of life. 

Although other research reports suggested that there is a relationship between a higher education level and anxiety during the pandemic [54], our findings suggest that lower education potentially may be a risk factor for greater sense of loss. Evidence suggesting that a lower level of education and lower socioeconomic status are associated with a higher level of anxiety, and a greater sense of loss during the pandemic has also been reported in other studies [44,69]. 

The association between lower education and anxiety may be linked to a lower willingness to seek knowledge on how to objectively perceive risks in daily life, how to counteract them, or how reformulate failures to one’s advantage [86,87]. A greater risk of experiencing high level of psychological distress due to COVID-19 is also faced by younger adults who continue their education or training [31].

### Limitations

Our study is not free of certain shortcomings. Due to the small numbers of respondents representing some nationalities it was impossible to consider this variable in the analyses. The majority of the participants were residents of Poland, Ukraine, United Kingdom, and Brazil. However, in other international studies related to strategies of coping with COVID-19 trauma, the variables related to the specific countries explained a very small percentage of outcomes (less than 7% in each case) [8]. Hence, the findings appear to be primarily linked to the generally difficult pandemic situation irrespective of the nationality and place of residence [51]. 

Furthermore, young adults constituted a majority of the respondents, which precluded assessment of the role of age as a predictor of negative perception of changes in various areas of life. We did not use an offline survey, and thus our study did not include people without internet access, a problem more commonly affecting the elderly. Due to this, our sample is not representative for older people. Moreover, a high rate of females registered for the study, and that was a limitation for our conclusions regarding any sex-related differences. In further research, it would be worthwhile to consider variables other than those considered in the present study—for instance, the participants’ personality traits since they determine biological predispositions for interpreting various events, including crisis situations, in specific ways. 

Another interesting approach would be to assess the sense of gains and losses resulting from the experience of pandemic relative to the baseline level of optimism, hope, and sense of meaning in life. It would also be interesting to further discuss the impact of the COVID-19 pandemic on the mental health and sense of changes in life in more fragile populations, such as pregnant women and patients recovered from more severe COVID-19 disease [88,89], due to their specific vulnerability to the negative impact of pandemic on their lives. 

In future research, more detailed information concerning participant lifestyles and changes in their habits (e.g., diet, participation in sport, and addictive behaviours) should be taken into consideration to better comprehend the complex underpinnings of subjective evaluation of one’s own life during the pandemic [90]. Moreover, comparisons between hospitalized and non-hospitalized COVID-19 recovered patients with regard to their sense of gains and losses during lockdown is another interesting area of future study [89].

## 5. Conclusions

The findings show that the perception of changes reported by individuals of various nationalities is not one-sided. In some areas of life, the opinions were rather positive and, in others, negative. Generally, there was a perception that the changes are negative; however, there was a group of individuals that perceived these as positive. Many factors contributed to such attitudes towards life during the pandemic; these included age and education. With age, there was a growing sense of a poorer relationship with parents and attention to health. 

A lower level of education resulted in a more negative perception of change in areas, such as the relationship with a partner, physical and mental health, work, activity for others, and the future after the pandemic. If a negative perception of changes resulting from the pandemic were accompanied by a higher degree of hyperarousal and intrusion, a more profound sense of loss was felt in various areas of life—in particular, related to mental and physical health, work, finances, intellectual capacities, activity for others (supporting relatives, volunteering, and involvement in non-governmental organizations), cultivation of a hobby, attention to politics, and daily life. An interesting conclusion is related to respondents with positive perceptions about the changes since they also present symptoms of intrusion and hyperarousal; however, these appear to be entangled with thinking and behaviour in a different way. 

It is likely that individuals perceiving change in positive terms are able to transform negative emotions, and this may lead to personal growth. These respondents in particular reported positive changes in relations with parents and in daily life during the pandemic, although they also noticed adverse effects in relations with their partner and in their work. It appears that intrusions and hyperarousal in some people play a role in structuring and motivating efforts to protect against the effects of the COVID-19 pandemic, in medical, social, and psychological dimensions. In others, these symptoms lead to frustration and anxiety adversely affecting the quality of life and may provide a false sense of security that is induced by anxiety.

## Figures and Tables

**Table 1:** Socio-demographic characteristics of the sample (*N* = 492).

Variables	Frequency	Percentage
Sex		
-female	362	73.6
-male	130	26.4
Education (level)		
-lower (no education/incomplete primary/complete primary/secondary)	208	42.3
-higher (university degree/PhD)	284	57.7
Occupational involvement		
-working people and/or university students	474	96.3
-pensioners, retirees, unemployed	18	3.7
Marital status		
-living with someone	431	87.6
-living on their own	61	12.4
COVID-19 history		
-had the disease, incl.:	149	30.3
stayed at home	146	29.7
stayed in hospital—mild case	2	0.4
stayed in hospital—severe case	1	0.2
-did not have the disease	343	69.7
Vaccination		
-fully or partly vaccinated individuals	340	69.1
-unvaccinated individuals	152	30.9
COVID-19 in relatives		
-yes	245	49.8
-no	247	50.2
Health status		
-chronic conditions present	39	7.9
-mild problems	153	31.1
-no medical conditions	300	61
Financial status		
-same as average	415	84.3
-significantly poorer	37	7.5
-significantly better	40	8.1
Nationality		
-Polish	260	52.8
-Ukrainian	109	22.2
-English	28	5.7
-Brazilian	70	14.2
-other	25	5.1

**Table 2:** Scores in the Impact of Event Scale: Revised (IES-R; *N* = 492; total sample).

IES-R	M (SD)
Intrusion (mean)	1.03 (0.68)
Avoidance (mean)	1.24 (0.68)
Hyperarousal (mean)	1.11 (0.69)
total IES-R (raw scores)	24.81 (12.87)

**Table 3:** Scores in the Sense of Life Changes Questionnaire (COVID-19 SLCHQ; *N* = 492; total sample).

Sense of Change in	M (SD)	*N* (%)
*relations with spouse/partner*	3.82 (1.54)	365 (74.2)
*relations with children/grandchildren*	4.02 (1.65)	252 (51.2)
*relations with parents*	4.21 (1.45)	419 (85.2)
relations with friends	3.83 (1.36)	492 (100)
*relations with co-workers*	3.99 (1.33)	353 (71.7)
mental health	3.49 (1.36)	492 (100)
physical health	3.47 (1.44)	492 (100)
*work*	3.73 (1.59)	361 (73.4)
finances	3.57 (1.37)	492 (100)
intellectual capacities	3.75 (1.37)	492 (100)
activity for others	3.42 (1.41)	492 (100)
attention to health	4.28 (1.47)	492 (100)
*religion*	4.07 (1.49)	366 (74.4)
hobby	4.04 (1.58)	492 (100)
attention to politics	3.81 (1.42)	492 (100)
daily life	3.21 (1.35)	492 (100)
future after the pandemic	3.90 (1.50)	492 (100)
total *	40.75 (10.014)	492 (100)

* The overall index was calculated based on 11 out of 17 subscales. The items excluded (marked with italic font) were those in which the answers provided accounted for less than 100%.

**Table 4:** Characteristics of participants with negative (NCH) and positive (PCH) sense of changes.

Variables	NCH (*n* = 362)	PCH (*n* = 130)	t/*p*-Value/
	M (SD)	M (SD)	Cohen’s d
intrusion IES-R	1.03 (0.69)	1.03 (0.64)	0.04/0.97
hyperarousal IES-R	1.15 (0.70)	1.01 (0.64)	−1.95 */0.05/0.21
avoidance IES-R	1.25 (0.68)	1.21 (0.69)	−0.52/0.60
total IES-R	25.09 (13.04)	24.04 (12.42)	−0.79/0.43
Age	34.40 (13.17)	33.41 (13.18)	−0.78/0.46
	*N*	*N*	χ^2^/*p*-value
Sex			
-female	91	1.16/0.28	
-male	91	39	
Education			
-lower	160	48	
-higher	202	82	2.07/0.15
Occupational involvement—working people and/or university students	347	127	
-pensioners, retirees, unemployed	15	3	0.91/0.34
Residence			
-living with another person	320	111	
-living on their own	42	19	0.80/0.37
Experience of COVID-19			
-yes	109	40	
-no	253	90	0.02/0.88
Vaccination			
-yes	251	89	
-no	111	41	0.03/0.85
COVID-19 in relatives			
-yes	185	60	
-no	177	70	0.94/0.33
Nationality			
-Polish	200	60	
-Ukrainian	72	37	
-English	22	6	
-Brazilian	54	16	
-other	14	11	9.46/0.06

* *p* ≤ 0.05.

**Table 5:** Correlations between the scores of the Impact of Event Scale: Revised (IES-R) and Sense of Life Changes Questionnaire (COVID-19 SLCHQ) in the groups with a negative (NCH) and positive (PCH) sense of changes.

Sense of Change in	IES-R Total r (*p*-Value)	Avoidance r (*p*-Value)	Intrusion r (*p*-Value)	Hyperarousal r (*p*-Value)
*relations with spouse/partner*	PCH −0.17 (0.04) *		PCH −0.252 (0.004) **	
*relations with children/grandchildren*				NCH −0.131 (0.04) *
*relations with parents*	PCH −0.16 (0.04) *		PCH 0.202 (0.014) *	
relations with friends			NCH −0.09 (*p* = 0.04) *	
mental health	NCH −0.197 (0.001) ***		NCH −0.185 (0.001) ***	NCH −0.221 (0.001) ***
physical health	NCH −0.169 (0.001) ***	NCH −0.12 (0.012) *	NCH −0.15 (0.003) **	NCH −0.165 (0.001) ***
*work*	NCH −0.143 (0.012) * PCH −0.227 (0.009) **	NCH −0.122 (0.03) *	PCH −0.35 (0.001) ***	NCH −0,19 (0.001) *** PCH −0.261 (0.003) **
Finances	NCH −0.102 (0.03) *	NCH −0.09 (0.049) *		NCH −0.138 (0.004) ** PCH −0.147 (0.047) *
intellectual capacities	NCH −0.144 (0.003) **		NCH −0.100 (0.03) *	NCH −0.222 (0.001) ***
activity for others	NCH −0.165 (0.001) ***		NCH −0.136 (0.005) **	NCH −0.222 (0.001) ***
Hobby	NCH −0.107 (0.02) *	NCH −0.122 (0.01) **		
attention to politics	NCH −0.169 (0.001)	NCH −0.09 (0.04) *	NCH −0.152 (0.002) **	NCH −0.199 (0.001) ***
daily life	NCH −0.154 (0.002) ** PCH 0.17 (0.026) *	NCH −0.087 (0.05) *	NCH −0.176 (0.001) *** PCH 0.155 (0.04) *	NCH −0.123 (0.01) ** PCH 0.152 (0.04) *
future after the pandemic	NCH −0.107 (0.02) *	PCH −0.195 (0.013) *	NCH −0.144 (0.003) **	NCH −0.11 (0.019) *

* *p* ≤ 0.05, ** *p* ≤ 0.01, and *** *p* ≤ 0.001; The variables analysed taking into account the missing data are marked in italics.

**Table 6:** Logistic regression analysis (enter method with likelihood ratio) associated with factors possibly related to sense of negative changes in life caused by the pandemic.

Model	B	Wald χ^2^	*p*-Value	OR [95% CL]	Lower [95% CL]	Upper [95% CL]
gender (1)	−0.263	1.259	0.262	0.769	0.485	1.217
age	0.017	2.908	0.07	1.017	0.997	1.038
level of education (1)	−0.435	3.064	0.08	0.648	0.398	1.053
personal experience of COVID (1)	0.083	0.130	0.718	1.087	0.692	1.708
COVID in relatives (1)	−0.124	0.332	0.564	0.883	0.580	1.346
Intrusion	−0.073	5.960	0.015 *	0.929	0.877	0.986
hyperarousal	0.145	9.645	0.002 **	1.156	1.055	1.266
Avoidance	0.001	0.001	0.971	1.001	0.949	1.056
type of occupational involvement (1)	0.239	0.121	0.728	1.270	0.330	4.891
vaccination status (1)	−0.021	0.008	0.928	0.979	0.615	1.559

* *p* ≤ 0.05, ** *p* ≤ 0.01; OR—odds ratio; variables coded as: vaccination status: yes = 0, no = 1; level of education: lower = 0, higher = 1; personal experience of COVID-19: yes = 0, no = 1; COVID-19 in relatives: yes = 0, no = 1; type of occupational involvement: working people and students = 0, pensioners, retirees, unemployed =1; gender: female = 0, and male = 1.

## Data Availability

The data that support the findings of this study are available from the corresponding author upon request.

## Appendix A

**Table A1:** The Impact of Event Scale: Revised (IES-R), and Sense of Life Changes Questionnaire (COVID-19 SLCHQ) scores—the comparisons with regard to different variables.

Variables	COVID-19 SLCHQ M (SD) t or F/*p*	Intrusion IES-R M (SD) t or F/*p*	Hyperarousal IES-R M (SD) t or F/*p*	Avoidance IES-R M (SD) t or F/*p*	IES-R Total Raw Score M (SD) t or F/*p*
Sex -female -male	40.65 (10.02) 41.04 (10.03) −0.38/0.71	1.15 (0.67) 0.99 (0.71) 0.78/0.43	1.13 (0.69) 1.06 (0.68) 1.12/0.27	1.27 (0.67) 1.15 (0.70) 1.68/0.09	25.29 (12.62) 23.48 (13.52) 1.37/0.17
Vaccination -yes -no	40.58 (9.31) 41.14 (11.46) −0.53/0.59	1.03 (0.69) 1.03 (0.65) 0.07/0.94	1.11 (0.70) 1.12 (0.67) −0.19/0.85	1.14 (0.66) 1.46 (0.69) −4.49/0.001 ***	24.02 (13.03) 26.58 (12.37) −2.03/0.04 *
COVID-19 history -yes -no	41.17 (9.92) 40.57 (10.06) 0.61/0.54	1.07 (0.70) 1.01 (0.67) 0.93/0.355	1.18 (0.71) 1.08 (0.68) 1.47/0.14	1.23 (0.72) 1.25 (0.67) −0.254/0.80	25.51 (13.91) 24.50 (12.40) 0.79/0.43
Education -lower -higher	39.40 (10.65) 41.75 (9.42) −2.58/0.011 *	1.03 (0.65) 1.21 (0.71) −0.008/0.99	1.21 (0.63) 1.05 (0.72) 2.62/0.009 **	1.26 (0.67) 1.22 (0.69) 0.59/0.55	25.57 (11.75) 24.25 (13.64) 1.18/0.26
COVID relatives -yes -no	40.41 (10.05) 41.09 (9.99) −0.74/0.45	1.07 (0.72) 0.99 (0.64) 1.15/0.25	1.17 (0.74) 1.06 (0.63) 1.75/0.08	1.23 (0.67) 1.25 (0.69) 0.42/0.67	25.29 (13.61) 24.34 (12.12) 0.81/0.42
Occupational involvement -working people, university students -pensioners, retirees, unemployed	40.84 (10.09) 38.39 (7.38) 1.021/0.31	1.02 (0.67) 1.17 (0.89) −0.911/0.36	1.11 (0.67) 1.23 (1.08) −0.74/0.46	1.24 (0.68) 1.35 (0.77) −0.72/0.47	24.70 (12.62) 27.61 (18.63) −0.66/0.52
Marital status -living with someone -living on their own	40.69 (9.95) 41.23 (10.52) −0.396/0.69	1.02 (0.67) 1.06 (0.77) −0.413/0.68	1.12 (0.69) 1.10 (0.69) 0.157/0.87	1.25 (0.68) 1.16 (0.73) 0.929/0.35	24.86 (12.75) 24.43 (13.84) 0.249/0.80
Health status -chronic conditions present -mild problems -no medical conditions	41.33 (10.13) 40.51 (9.18) 40.80 (10.43) 0.114/0.82	1.04 (0.83) 1.11 (0.75) 0.99 (0.62) 1.563/0.211	1.02 (0.75) 1.23 (0.75) 1.07 (0.64) 3.37 ^1^ (0.05) *	1.14 (0.66) 1.30 (0.71) 1.22 (0.67) 1.114/0.33	23.59 (14.33) 26.63 (14.08) 24.05 (11.95) 2.22/0.11
Financial status -same as average -significantly poorer -significantly better	40.9 (9.82) 37.73 (9.94) 42.03 (11.69) 2.063/0.128	1.01 (0.66) 1.03 (0.83) 1.19 (0.78) 1.178/0.309	1.09 (0.66) 1.35 (0.91) 1.15 (0.69) 2.505/0.08	1.25 (0.68) 1.24 (0.73) 1.17 (0.73) 0.238/0.788	24.59 (12.47) 26.30 (15.86) 25.73 (14.15) 0.408/0.665

* *p* ≤ 0.05, ** *p* ≤ 0.01, *** *p* ≤ 0.001; ^1^ mild problems > no medical conditions; *p* = 0.05 (post-hoc; the Bonferroni test).
